# Perinatal outcomes of hypertensive disorders in pregnancy at a referral hospital, Southern Ethiopia

**DOI:** 10.1371/journal.pone.0213240

**Published:** 2019-02-28

**Authors:** Netsanet Abera Asseffa, Birhanu Wondimeneh Demissie

**Affiliations:** College of Health Sciences and Medicine, Wolaita Sodo University, Sodo, Ethiopia; Liverpool School of Tropical Medicine, UNITED KINGDOM

## Abstract

**Introduction:**

Hypertensive Disorders in Pregnancy (HDP) complicate about 10% of pregnancies. It accounts to 50% of maternal death in sub-Saharan Africa and precedes 15% of perinatal deaths worldwide. In this study, we looked at the perinatal outcomes and factors associated with unfavorable perinatal outcomes among women with hypertensive disorders in pregnancy at Wolaita Sodo teaching and referral hospital, southern Ethiopia.

**Methods:**

A hospital based retrospective cross-sectional study design was employed among women hospitalized for hypertensive disorders in pregnancy. Socio-demographic, obstetrics, clinical laboratory, pregnancy complications and outcome were checked from patient records. Descriptive statistics were used to describe parameters collected from patient records. Bivariate and multiple logistic regressions were done to determine factors associated with unfavorable perinatal outcome. A P-value of less than 0.05 and 95% confidence interval not including 1 were considered statically significant.

**Results:**

There were 168 (2.3%) cases of HDP of the total 7, 347 deliveries during the period of the study from January 2014-December 2016. 72.5% of mothers (72.5%) had vaginal delivery and 26.1% had Caesarean Section. This study revealed a perinatal mortality rate of 111.1 per 1000 live births. On bivariate logistic regression variables such as referral status, diastolic blood pressure, ANC use, types of HDP, fetal weight at birth, maternal complication and maternal outcome were found to be associated with unfavorable perinatal outcomes. On multiple logistic regression fetal weight at birth and maternal outcome were found to be an independent predictors of unfavorable perinatal outcome.

**Conclusion:**

Our study shows higher perinatal mortality in a tertiary hospital where emergency obstetric and newborn care is set and quality obstetric care is expected. However, tertiary facilities manage difficult cases which can explain the high PMR. But it is high which means there is enough room for improvement. Hence, the referral hospital and neighboring health facilities should give due emphasis for early detection and management of women with HDP.

## Introduction

Hypertensive Disorders in Pregnancy (HDP) complicate about 10% of pregnancies with increasing wealth and non-communicable risk factors for HDP; it will increase the incidence of HDP. HDP account for 50% of maternal death in sub-Saharan Africa [1–3]. It has been estimated that the HDP precedes 15% of perinatal deaths worldwide [4]. Moreover, 73% of newborn deaths occur within first seven days of birth [5]; showing that the greatest burden of preventable mortality occurs days after birth. Perinatal mortality is the total of number of stillbirths and deaths occurring within the first week of life. It is among the most sensitive indicators of obstetric care [2,6].

It has been pointed out that women with HDP may experience various complications, adverse outcomes to the fetus and mortality. Hypertensive disorders in pregnancies are associated with fetal growth restriction, perinatal asphyxia, iatrogenic prematurity, stillbirths, preterm delivery, perinatal death, neonatal mortality and affects vital maternal organ system such as renal, hepatic, cardiorespiratory, fetoplacental and hematologic [7]. Studies showed that about 30,000 maternal deaths [7], 30% of maternal near-miss events [8,9], 16% of 2.6 million stillbirths [10], 10% perinatal deaths (8/1000live births) are associated with HDP [11]. The primary objective of HDP treatment is to prevent potential maternal complications and death whose importance to the fetus is dubious. Moreover the risk of perinatal death from hypertensive disorders in pregnancy is more daunting than the maternal death. For instance, the risk of perinatal death from severe pre-eclampsia is 13% whereas maternal death is less than 1% let-alone the short and long term consequences in surviving new-born [12,13]

Worldwide, 6.3 million perinatal deaths occur every year [14]. As in many developing countries, Ethiopia has a high burden of perinatal death (46per 1000 births) [15]; this is a national survey data however in some regions this rate is higher. For instance, a study conducted in central Ethiopia showed 72 per 1000 births [16] where facility based childbirth is highest. Outrageously a study conducted four decades ago showed a similar rate of perinatal death; telling very little progress has been made [17]. This proves that neither type of attendance nor place of childbirth found to lower perinatal mortality but early detection and treatment with good quality of care [18]. In the nation, the leading causes of perinatal deaths are hypertensive disorders of pregnancy, obstructed labour, fetal mal-presentation and uterine rupture. In this context, exploring perinatal outcomes at facility level will be important to inform programmers to design interventions for better perinatal outcomes. In this study, we looked for factors associated with unfavorable perinatal outcome among women with hypertensive disorders in pregnancy at Wolaita Sodo teaching and referral hospital, southern Ethiopia.

## Methods

### Study design, setting and population

This hospital based retrospective cross-sectional study was conducted from 1^st^ January 2014-31^st^ December 2016 in Wolaita Sodo Teaching and Referral Hospital. The hospital has served since 1928; 50 years as primary hospital and33 years as district hospital. It served as a referral center since six years for a catchment area with a population of approximately three million inhabitants. The tertiary hospital has 688 health professionals of which four are obstetricians and gynecologist and 215 are midwives and nurses. It had a total of 250 beds of which 37 were Obstetrics & Gynecology ward beds and had three delivery coaches [19].

### Measurement tools and data collection

Three trained medical interns collected the data from medical records of women hospitalized for HDP and compared this with the Health Management Information System (HMIS) registry to confirm accuracy of the data. And those reliable were included in the study; eight charts were not found in the HMIS registry. All pregnant mothers who have HDP and gave birth after 28weeks of gestation at Wolaita Sodo Teaching and Referral Hospital over the study period were included. We excluded women with twin gestations and congenital birth defects to avoid their potential confounding effect on the unfavorable perinatal outcomes associated with HDP. Charts with incomplete data were also excluded from the study.

A structured data collecting format was prepared after reviewing relevant literature and used to abstract relevant data from the included patients’ charts. Socio-demographic data checked from the files were maternal age, address and source of referral. Obstetric characteristics obtained were history of stillbirth, gestational age, parity, number of gestation, onset of HDP, antenatal care visit, birth weight and diagnosis at admission. Other clinical and laboratory data were systolic blood pressure, diastolic blood pressure, platelet count, liver and renal function results. Pregnancy complications and outcomes were onset of labour, treatment given, mode of delivery, birth weight, maternal complication, maternal outcome and fetal outcome S1 Questionnaire.

### Operational definitions

Hypertension in pregnancy was defined as systolic blood pressure (BP) ≥140 mmHg and/or a diastolic BP ≥90 mmHg. Preeclampsia was defined as characterized by a BP of 140/90 mm Hg or greater after 20 weeks’ gestation in a women with previously normal BP and who have proteinuria. Eclampsia was defined as seizures that cannot be attributable to other causes, in a woman with preeclampsia. In this study, hypertensive disorders of pregnancy were classified as preeclampsia, gestational hypertension, chronic hypertension and preeclampsia superimposed on chronic hypertension. Low birth weight was defined as birth weight of 2.5kg or less, regardless of gestational age. Preterm delivery is any birth before 37 weeks completed weeks of gestation. Unfavorable perinatal outcome stands for those admitted to neonatal intensive care unit and/or death [20,21].

### Data analysis

After completeness was checked, data were coded and entered into EpiData version 3.1 statistical software and exported to SPSS version 20 statistical software for analysis. We used descriptive statistics to describe parameters collected from the files. Bivariate and multiple logistic regression were done to determine factors associated with unfavorable perinatal outcome. Variables which did not show statistical significance in the bivariate analysis were excluded from the multivariate analysis. *P*-value less than 0.05 and 95% confidence interval not including 1 were considered statically significant.

### Ethical consideration

Ethical approval was obtained from Wolaita Sodo University, College of Health sciences and Medicine ethical review committee. Regarding informed consent, the ethics committee waived the requirement for informed consent; however confidentiality was maintained.

## Results

### Socio-demographic characteristics of participants

There were 168 (2.3%) cases of HDP of the total 7, 347 deliveries during the period of the study from January 2014to December 2016. Out of the total, 15 were excluded from the analysis due to incompleteness, twin gestation and/or congenial birth defects. The mean age of mothers during the study period was 25.42 ±4.8SD. Most of the participants, 86 (56.2%) were rural residents. Of the total HDP cases, 135 (88.2%) were referred from neighboring health facilities “Table 1”.

**Table 1:** Characteristic of women diagnosed with HDP at Wolaita Sodo teaching and referral hospital, Southern Ethiopia.

Variables	N	%
Residence		
Rural	86	56.2
Urban	67	43.8
Source of referral		
Yes	135	88.2
No	18	11.8
Past Medical history		
Chronic Liver Disease	1	0.65
Diabetes Mellitus	2	1.3
Chronic Hypertension	1	0.65
No history medical illness	149	97.4
Age		
<25	58	37.9
25–30	58	37.9
>30	37	24.2
**Parity**		
0	76	49.7
1–2	52	33.9
>3	25	16.4
ANC* attendance		
Yes	134	87.6
No	19	12.4
**Gestational age at birth**		
Preterm	43	28.1
Term	110	71.9
Post-term	0	0
**Mode of delivery**		
SVD*	111	72.5
Instrumental	2	26.1
Cesarean section	40	1.3
**Birth weight**		
Normal birth weight	138	90.2
Low birth weight (<2.5kg)	15	9.8
**Diagnosis at admission**		
Mild preeclampsia	32	20.9
Severe Preeclampsia	77	50.3
Eclampsia	40	26.1
Super imposed preeclampsia	4	2.6
**Perinatal Outcome**		
Alive during discharge	119	77.8
NICU	17	11.1
Death	17	11.1

ANC* Antenatal Care, SVD* Spontaneous Vaginal Delivery

### Obstetrics and clinical characteristics of participants

Of the total participants, 76(49.7%) were primigravida and 87.6% had at least one ANC contact. The proportion of preterm delivery was 28.1% whereas low birth weight was 9.8%. A total of 47 (30.7%) had Systolic Blood Pressure of 160mmhg or more and 38 (24.8%) of them had Diastolic Blood Pressure of 110mmhg or more. The prevalence of severe preeclampsia, eclampsia, mild preeclampsia and super imposed preeclampsia were 77 (50.3%), 40 (26.1%), 32 (20.9%), and 4 (2.6%) respectively. The majority of mothers (72.5%) had Spontaneous Vaginal Delivery (SVD) and 26.1% had Caesarean Section “Table 1”. Concerning the onset of HDP, 148(96.7%) occurred during the antepartum period and all mothers were treated with magnesium sulphate.

Regarding maternal complications, it was recorded in 69 (45.1%) of women with HDP; 30 (19.6%), 14 (9.2%), 13 (8.5%), 9 (5.9%), 2 (1.3%), and 1(0.7%) were eclampsia, Hemolysis, Elevated Liver enzymes syndrome, Acute Kidney Injury, Postpartum Hemorrhage, Disseminated Intravascular Coagulation, and pulmonary edema respectively. There was no perinatal death reported among those delivered by caesarean section. Of the total perinatal death, 64.7% were preterm and 35.3% had birth weight of less than 2.5kg. Concerning the type of HDP and complications developed, 84.5% of those with Acute Kidney Injury complications were diagnosed with severe preeclampsia. On the other hand, the mean length of postpartum hospital stay was 2.89 (SD = 0.74) days.

### Factors associated with unfavorable perinatal outcome

On bivariate logistic regression, variables such as referral, diastolic blood pressure, ANC use, fetal weight at birth, maternal complication and maternal outcome were found to be associated with unfavorable perinatal outcomes. On multiple logistic regression, fetal weight at birth and maternal outcome were found to be an independent predictors of unfavorable perinatal outcome “Table 2”.

**Table 2:** Bivariate and multiple logistic regression of perinatal outcomes of hypertensive disorders in pregnancy at referral hospital in Ethiopia, 2017.

	Perinatal Outcome		COR* (95% CI*)	*P*-value	AOR(95% CI*)	*P*-value
	Favorable n(%)	Unfavorable n(%)				
**Referral**						
Yes	109(80.7)	26(19.3)	Ref		Ref	
No	10(55.6)	8(44.4)	0.29(0.1–0.830)	0.02	1.89(0.4–7.0)	0.39
DPB*, mm Hg						
90–110	95(82.6)	20(17.4)	2.77(1.2–6.2)	0.01	0.7(0.2–2.4)	0.59
>110	24(63.1)	14(36.8)	Ref		Ref	
ANC* use						
Yes	108(80.6)	26(19.4)	3.02(1.1–8.2)	0.03	0.84(0.2–3.7)	0.86
No	11(57.9)	8(42.1)	Ref		Ref	
**Mode of delivery**						
SVD*	83(73.4)	30(26.5)	Ref		Ref	
Cesarean section	36(90.0)	4(10.0)	3.25(1.0–9.91)	0.03	2.3(0.7–7.4)	0.15
**Birth Weight**						
Normal Birth weight	113(81.9)	25(18.1)	6.78(2.2–20.7)	0.00	4.1(1.0–17.1)	0.04
Low Birth weight	6(40.0)	9(60.0)	Ref		Ref	
**Maternal Complication**						
Yes	48(69.6)	21(30.4)	Ref		Ref	
No	71(84.5)	13(15.5)	2.3(1.1–5.2)	0.02	0.7(0.2–1.8)	0.50
**Maternal Outcome**						
Alive on discharge	24(17.1)	116(82.8)	16.11(4.1–62.9)	0.00	11.0(1.8–66.0)	0.00
Dead	10(76.9)	3(23.0)	Ref		Ref	

ANC*- Antenatal Care, AOR*- Adjusted Odds Ratio, CI*- Confidence Interval, COR*- Crude Odds Ratio, DBP*- Diastolic Blood Pressure, SVD* -Spontaneous Vaginal Delivery. As the instrumental delivery was only 2 it was merged to SVD for logistic regression. *p*-value significant at p<0.05.

## Discussion

This retrospective study assessed perinatal outcomes and factors associated with unfavorable perinatal outcomes among women with hypertensive disorders in pregnancy. Out of the total 7347 deliveries, 168 (2.3%) had HDP of which 15 were excluded from the analysis due to either incompleteness or twin gestation. On binary logistic regression, factors such as referral, diastolic blood pressure, ANC use, diagnosis at admission and maternal outcome were associated with unfavorable perinatal outcome. On the final model, only birth weight and maternal outcome were found to be independent predictors of unfavorable perinatal outcome.

In this study, the magnitude of HDP was 2.3%; which is consistent with a study conducted in Dilla referral hospital, Ethiopia (2.2%) [22]. However, the finding is lower than a retrospective study conducted in the Ethiopian capital, Addis Ababa (4.3%) [23], this inconsistency might be related to better detection rate in the capital Addis Ababa where more than 50% nations specialist doctors live [24]. In addition, the finding of this study is lower than a meta-analysis done on prevalence of hypertensive disorders of pregnancy in Ethiopia (overall pooled prevalence = 6.07%) [25]. The difference might be related to the use of random effect analysis following wide-ranging of HDP prevalence (1.2% to 18.25%) in the nation.

Based on the results of this study, 20.9% were diagnosed with mild preeclampsia, 50.3% had severe preeclampsia, 26.1% had eclampsia, and the rest 2.6% had superimposed preeclampsia. The variability among the types HDP might be explained by the fact that women with pre-eclampsia or eclampsia have symptoms and are more easily detected than women with pregnancy induced hypertension who can only be detected by screening during visits. This finding is higher in all types of HDP as compared to the WHO multicountry survey on maternal and newborn health [11]. This variability might be due to the fact that the WHO study has been done in many health facilities (357 health facilities) and conducted in various parts of the world. Moreover, this might be related to the fact that most of the participants (87.6%) of this study were referred from other neighboring facilities showing there was some degree of complication before arrival. In this study, all women presented with HDP received MgSO4. This is contrary to a study done in Tanzania where all patients with eclampsia and most patients with pre-eclampsia received MgSO4. This variability might be explained by the fact that the hospitals have different treatment protocols [1]. However, a randomized controlled trial study showed that treating women with mild pre-eclampsia with MgSO4 is not always necessary [26].

This study revealed a perinatal mortality rate of 111.1 per 1000 live births. The perinatal mortality rate is higher as compared to studies done in Northern Tanzania, 57.7 per 1000 live births. The difference might be related to maternal complications which were observed in 45.1% of women with HDP. In addition, it might be related to the low birth weight in our study as it was an independent predictor of unfavorable perinatal outcome. Moreover, women visit a referral center when they cannot be managed at home or health center which deters early detection and treatment [1, 27–29].

In this study, maternal age has no significant association with unfavorable perinatal outcome. This finding is consistent with a retrospective hospital based studies done in Ethiopia and Ghana [2,30]. The findings of this study showed that those who were referred from neighboring health facilities had more unfavorable perinatal outcome as compared to those who directly visited the referral hospital. This might be related to delay in reaching the facility, decentralization of services, and/or weak referral linkage [31].

Regarding the antenatal care visit, women who had not had ANC attendance were three times more likely to have unfavorable perinatal outcome as compared to women who did not attend antenatal care (COR = 3.02, 95% CI [1.1–8.2]). This might due to the fact that ANC gives opportunities to deal with many conditions directly or indirectly related to pregnancy including the HDP. On the other hand, mothers who had normal fetal weight at birth had four times lower risk of unfavorable perinatal outcome as compared to those with low birth weight (AOR = 4.1, 95% CI [1.0–17.1]). This finding is in line with a study done in Nigeria [32]. This consistency might be related to the fact that in both studies there was higher percentage of severe preeclampsia contributing to low birth weight. Moreover, it is known that low birth weight is a major determinant of perinatal survival and intensive care admission [33,34].

On binary logistic regression, those who delivered by spontaneous vaginal delivery were three times more likely to have unfavorable perinatal outcome as compared to those who delivered spontaneously (COR = 3.25, 95% C.I [1.0–9.91]). This finding is comparable with retrospective study done in three hospitals in Ethiopia [34]. This might be related to early detection and management by the professionals. It is worth mentioning that in this study there was no perinatal death reported among those delivered by caesarean section. However, the maternal risk of caesarean section might have been high particularly among women with illness.

On multiple logistic regression, birth weight was found to be an independent predictor of unfavorable perinatal outcome. The proportion of unfavorable perinatal outcome was four fold higher in low fetal weight at birth than normal fetal weight at birth. This finding is comparable with several studies [2,23,34]. It might be related to the scientific fact that HDP is associated with intrauterine growth restriction and preterm deliveries [7]. This study showed that 49.0% of deliveries were preterm. The other independent predictor of unfavorable perinatal outcome was maternal outcome. The findings of this study showed that all women who arrived with HDP were treated with magnesium sulphate whose role in reduction of maternal death from HDP is well known. However, 13 (8.5%) women with HDP had died. The reason may need further research on both supply and demand side.

There are important limitations of this study that need to be taken into account when interpreting the findings reported. As the design was retrospective in nature, service quality and related factors could not studied, and hence could not be adjusted for in the statistical analyses. The study did not include control groups, possibly limiting the extent to which cause and effect can be attributed to the study findings. After adjustment for some of the potential explanatory factors considered, marked changes were observed between the unadjusted (COR) and adjusted (AOR) odds ratios suggesting that other confounding factors might be operating; however, the sample size available to this study was insufficient to support a more detailed statistical evaluation of this issue. Finally, the competency of the professionals responsible for collecting the study data varied considerably, raising issues about the accuracy of some diagnoses. Consequently, caution should be exercised when generalizing the findings of this study to other populations.

## Conclusion

Our study showed higher perinatal mortality in a tertiary hospital where emergency obstetric and newborn care is set and quality obstetric care is expected. However, it is worth to note that tertiary facilities manage difficult cases which can explain the high PMR. But it is high which means there is enough room for improvement. It has also showed that no perinatal death was reported among those delivered by caesarean section. Hence, the referral hospital and neighboring health facilities should give due emphasis for early detection and management of women with HDP. Moreover, maternal mortality audit to improve quality of obstetric care for every pregnant woman and her baby has to be stronger.

The findings of this study affirmed that that perinatal and maternal mortality are closely related and interventions to prevent maternal complications will benefit perinatal outcome as well. In this aspect, antenatal care service has the potential to improve the feto-maternal outcome as it gives the opportunity to assess risk factors, early detect any possible hypertensive disorders during pregnancy and improve health seeking behavior. Therefore, antenatal care should be given by practitioners with good clinical and interpersonal skills within a well-functioning health system.

## Supporting information

S1 Dataset (SAV)

S1 Questionnaire (DOCX)

## Abbreviations

AKI: Acute Kidney Injury
ANC: Antenatal Care
AOR: Adjusted Odds, Ratio
BP: Blood Pressure
CI: Confidence Interval
COR: Crude Odds Ratio
CS: Caesarean Section
DBP: Diastolic Blood Pressure
DIC: Disseminated Intravascular Coagulation
HDP: Hypertensive Disorders of Pregnancy
HELLP: Hemolysis, Elevated Liver enzymes, and Low Platelet count
NICU: Neonatal Intensive Care Unit
PPH: Postpartum Hemorrhage
SVD: Spontaneous Vaginal Delivery
WHO: World Health Organization
