# Supplementary material for: Perinatal outcomes of hypertensive disorders in pregnancy at a referral hospital, Southern Ethiopia
Source: PLoS One. 2019 Feb 28;14(2):e0213240. doi: 10.1371/journal.pone.0213240 (PMC6394918; doi:10.1371/journal.pone.0213240)
Supplement: S1 Questionnaire — (DOCX) [file pone.0213240.s002.docx]

**S1 (Questionnaire)**

| Section I: - Socio-demographic Characteristics | | | |
| --- | --- | --- | --- |
| No | Question | Response | Skip |
|  | Place of residence | Urban……………………1  Rural…………………….2 |  |
|  | Age | _________ (in years) ......................1 |  |
|  | Source of referral | Yes…………………………………………1  No………………………………………….2 |  |
|  | History of still birth | Yes…………………………………………1  No………………………………………….2 |  |
|  | Past Medical illness | ___________________________ |  |
|  | Gravidity | Primgravida ……………………………1  Multigravida …….…………………….2 |  |
|  | Parity | ______________________ in number |  |
|  | Gestational age | Preterm ……………………...…………...1  Term…………………….………………...2 |  |
|  | Number of foetus | Singleton…………………………………1  Multiple……………………………….….2 |  |
|  | ANC use | Yes…………………………………………1  No………………………………………….2 |  |
|  | History of admission during pregnancy | Yes…………………………………………1  No………………………………………….2 |  |
|  | Drugs used during pregnancy | Yes…………………………………………1  No………………………………………….2 |  |
|  | Type of HDP | Mild Preeclampsia……………………..1  Severe Preeclampsia ………………….2  Eclampsia………………………………3  Superimposed…………………………..4 |  |
|  | Onset of HDP | Antepartum ……………………………1  Postpartum ……………………….…….2 |  |
|  | Highest systolic BP | ________________________ |  |
|  | Highest diastolic BP | _________________________ |  |
|  | Platelet count | _________________________ |  |
|  | Liver function test | Normal………………………………1  Abnormal…….……………………….2  Twice…….……………………………3 |  |
|  | RFT | _____________________________ |  |
|  | On set of labour | Induced ………………………………1  Spontaneous…………………………….2 |  |
|  | Mode of delivery | SVD…………………………………..1  CS……..………………………………2  Others…………………………………3 |  |
|  | Birth weight | _____________________________ |  |
|  | Duration of admission | ___________________________ |  |
|  | Hospital stay after delivery | _____________________________ |  |
|  | Maternal complication | Yes . . . . . . . . . . . . . . . . . . . . . . . . . . . . . . . . . 1  No . . . . . . . . . . . . . . . . . . . . . . . . . . . . . . . . . 2 |  |
|  | Type of maternal complication | _________________________________ |  |
|  | Perinatal outcome | Alive on discharge…………………1  NICU………………………………….2  Died……………………………………3  Others specify_______________________ |  |
|  | Maternal outcome | Alive on discharge………………………1  Died.………………………..…………...2 |  |
